# An oxidative stress-related molecular signature in atherosclerosis: identification of risk genes, construction of a diagnostic model, and characterization of immunocyte landscape

**DOI:** 10.3389/fcvm.2025.1600321

**Published:** 2025-08-14

**Authors:** Zhile Li, Hong Ling, Qiuyu Wei, Xiukai Tang, Danyi Zhang, Zhaohe Huang

**Affiliations:** ^1^ Department of Cardiology, Affiliated Hospital of Youjiang Medical University for Nationalities, Baise, Guangxi, China; ^2^ Laboratory of the Atherosclerosis and Ischemic Cardiovascular Diseases, Affiliated Hospital of Youjiang Medical University for Nationalities, Baise, Guangxi, China

**Keywords:** oxidative stress, circadian rhythm, atherosclerosis, risk model, immune infiltration

## Abstract

Atherosclerosis (AS), the primary cause of cardiovascular disorders and stroke, is a complex, multifactorial disease. Numerous studies have shown that oxidative stress and circadian disruption are paramount contributors to the development of AS and its complications. Nevertheless, there is no applicable related diagnostic model to assess the AS clinical risk according to patients' oxidative stress status and circadian rhythm molecular expression. This study aimed to develop an oxidative stress–circadian rhythm-related model using AS cohorts (GSE100927 and GSE43292) to explore the potential relationship between AS and oxidative stress with circadian rhythm. We screened the significant oxidative stress–circadian rhythm-related genes in AS samples by integrating two datasets by various machine learning methods. Then, we developed an oxidative stress–circadian rhythm-related diagnostic model based on six risk genes (*IL1RN*, *CA2*, *PDE8B*, *RYR2*, *DPP4*, *TDO2*) identified through LASSO regression analysis and a nomogram diagram. Calibration and decision curve analysis (DCA) showed the relevant accuracy of the risk model. Receiver operating characteristic curve (ROC) delineated the higher reliability of our model than each single risk gene diagnostic model. Then, we verified the accuracy of our model in the validation dataset (GSE27034). Latent regulatory networks (including miRNA, transcription factor, and small-molecule compound) regarding risk genes were also constructed using the ENCORO, ChIPBase, and CTD databases. We observed significantly greater immune infiltration in the high-risk group of AS samples than that in the low-risk group based on the linear predictor derived from our logistic model. Finally, we classified the AS samples into two subtypes according to the expression patterns of risk genes and, interestingly, found an obvious discrepancy in immune cell infiltration between these subtypes.

## Introduction

Cardiovascular disease, the leading cause of death worldwide (1), mainly arises from atherosclerosis (AS) (2), a chronic inflammatory condition of the arteries characterized by the accumulation of lipids and inflammatory cells in the arterial walls (3). Normally, AS begins with endothelial injury caused by various factors at the position with altered shear stress (4). Low-density lipoprotein cholesterol (LDL) and inflammatory cells penetrate the injured endothelium and accumulate in the subendothelial space. The accumulated LDL undergoes oxidation by reactive oxygen species (ROS) or other oxidation factors (5), and the oxidized LDL acts as the core to promote the expression of cell adhesion molecules on endothelial cells and promote the recruitment of circulating monocytes into the subendothelial space (6, 7). The recruited monocytes differentiate into macrophages and engulf LDL. As LDL accumulates, macrophages transform into lipid-laden foam cells that release inflammatory factors to further assemble more monocytes or other inflammatory cells (6). Finally, accompanied by smooth muscle cell migration and extracellular matrix stacking, atherosclerotic plaques develop (8).

Oxidative stress is a significant factor in the progression and development of atherosclerosis (9). The most important oxidative stress-related factors in atherosclerosis are ROS, including free radical species such as superoxide (O_2_·^−^) and hydroxyl species (HO·), and non-free radicals such as hydrogen peroxide (H_2_O_2_) (10, 11). The major role of ROS in atherosclerosis is the oxidation of LDL to form oxidized LDL, which is the core of atherosclerotic plaque formation (12). At the onset of atherosclerosis, endothelial cells are the main source of ROS (13). Due to NO reduction or consumption in endothelial cells, ROS production is further amplified (14). Additionally, ROS can induce endothelial cells to become proinflammatory cells, indicating that endothelial cells are not only a source but also a target of ROS (15). These proinflammatory cells are indispensable for the development of atherosclerosis through inducing directional migration of monocytes (16). In addition, ROS is associated with the stability of atherosclerotic plaques (17). Matrix metalloproteinases (MMP) are involved in the maintenance of atherosclerotic plaques by degrading extracellular matrix (ECM) collagen, a major component of the protective cap of plaques (18). ROS indirectly upregulate MMP expression by inducing oxidized LDL accumulation (19, 20). Therefore, ROS plays a role in all stages of atherosclerosis.

An increasing amount of evidence reveals that AS pathogenesis demonstrates intimate connections with circadian regulatory mechanisms, particularly through the modulation of lipid metabolism, oxidative stress, inflammatory responses, and endothelial homeostasis (21, 22). Circadian rhythm disruption elevates LDL levels and impairs HDL functionality, exacerbating lipid deposition in arterial walls (23). Core clock genes, such as BMAL1 and CLOCK, govern vascular redox balance by transcriptional regulation of antioxidant enzymes such as superoxide dismutase (SOD) and catalase (CAT), thereby modulating reactive oxygen species (ROS) scavenging capacity (24, 25). The circadian nuclear receptor Rev-erb α attenuates vascular inflammation by suppressing NF-κB–mediated polarization of macrophages toward proinflammatory M1 phenotypes (26). Endothelial PER2 oscillation rhythmically regulates nitric oxide (NO) bioavailability through posttranslational modification of endothelial NO synthase (eNOS), critically influencing vasomotor function (13). Notably, some of the oxidative stress–circadian rhythm cross-talk genes (HMOX1, CD36, IL1B) show significant enrichment in atherosclerotic plaques, suggesting their dual regulatory roles may represent novel therapeutic targets (27–29). HMOX1 modulates heme catabolism and oxidative defense, while CD36 coordinates circadian lipid uptake rhythms and foam cell formation.

Recently, all kinds of bioinformatic methods have been used to reveal potential molecular biological processes in various diseases based on high-throughput sequencing and microarray data (30–32). In this research, we used two public datasets and different bioinformatic analysis methods to explore the relationship between oxidative stress–circadian rhythm-related genes (CRGs) and AS and developed a clinical risk model to assist clinicians in diagnosing AS and identifying immune cell infiltration in atherosclerosis with a molecular background that may contribute to the discovery of new therapeutic targets for AS management.

## Methods and materials

### Data download and integration

The atherosclerosis (AS) datasets (GSE100927, GSE43292) and the peripheral arterial disease (PAD) dataset (GSE27034) were downloaded from the GEO database (https://www.ncbi.nlm.nih.gov/geo/) using the R package GEOquery. Circadian rhythm-related genes (CRGs) and oxidative stress-related genes (ORGs) were obtained from the GeneCards database (https://www.genecards.org/) and published literature, respectively (33–35). Batch effects between GSE100927 and GSE43292 were removed using the R package sva to generate an integrated GEO dataset (designated as the testing dataset), while GSE27034 served as the validation dataset. The merged dataset underwent normalization, probe annotation, and standardization using the *limma* package. Principal component analysis (PCA) was performed on expression matrices before and after batch correction to validate the efficacy of batch effect removal.

### Identification of oxidative stress–circadian rhythm-related differentially expressed genes

Differential expression analysis between atherosclerotic (AS) and control samples in the integrated dataset was performed using the R package *limma*. Genes with |logFC| > 1.0 and *p* < 0.05 were defined as differentially expressed genes (DEGs), where upregulated genes met logFC > 1.0 and *p* < 0.05 and downregulated genes met logFC < −1.0 and *p* < 0.05. The intersection between DEGs, oxidative stress-related genes, and circadian rhythm-related genes was identified through Venn diagram analysis, yielding oxidative stress–circadian rhythm-related differentially expressed genes (OCRDEGs). Visualization of results, including volcano plots and Venn diagrams, was conducted using the R package *ggplot2*.

### Functional and pathway enrichment analysis

Gene Ontology (GO) and KEGG pathway enrichment analysis were performed for OCRDEGs using the R package *clusterProfiler*, with biological terms filtered at thresholds of *p* < 0.05 and false discovery rate (FDR) of *q* < 0.25. Gene set enrichment analysis (GSEA) was conducted on all DEGs in the integrated GEO dataset (combined datasets) using *clusterProfiler*, with gene sets (c2.all.v2024.1.Hs.symbols.gmt) obtained from the Molecular Signatures Database (MSigDB) [all canonical pathways, *n* = 3,050]. GSEA results were filtered using adjusted *p* < 0.05 and FDR *q* < 0.25.

### Identification of core genes and construction of a logistic regression model

Core OCRDEGs were screened using three independent machine learning approaches: random forest, LASSO regression, and support vector machine-recursive feature elimination (SVM-RFE). Regarding parameter settings, the ntree value for the random forest model was set to 1,000 to ensure the model's stability and convergence. In LASSO regression, we automatically selected the optimal *λ* value through cross-validation, and when analyzing the differentially expressed genes related to oxidative stress–circadian rhythm (OCRDEGs), we performed LASSO regression with family = “binomial” as a parameter; the number of features in SVM-RFE was determined based on the minimum error rate. Intersection genes from these methods were designated as key risk genes (key genes). These key genes underwent further logistic regression filtering (significance threshold: *p* < 0.05) to identify hub genes (hub genes), with risk associations visualized via forest plots using the forestplot package. A multivariate logistic regression model was constructed using hub genes, and diagnostic performance was visualized through a nomogram. Model accuracy and clinical utility were validated using calibration curves and decision curve analysis (DCA). Friends analysis among hub genes was assessed by the GOSemSim R package, and Spearman correlation coefficients among hub genes were calculated. Chromosomal localization of hub genes was mapped using the RCircos package.

### Validation of hub gene differential expression and ROC curve analysis

Differential expression analysis of hub genes was performed in both the training dataset and the validation dataset. A multivariate logistic regression model constructed based on hub genes from the training set was employed to calculate linear predictors in the training set. The formula was:

$$\mathrm{Linear}\;\;\mathrm{Predictor}\;=\;\sum_{i}\mathrm{Coefficient}\;(\mathrm{gen}e_{i})*\mathrm{mRNA}\;\mathrm{Expression}\;(\mathrm{gen}e_{i})$$

The R package pROC was utilized to plot receiver operating characteristic (ROC) curves and compute the area under the curve (AUC) values, evaluating the diagnostic capacity of hub genes for AS. Additionally, linear predictors in the validation dataset GSE27034 were calculated according to the diagnostic formula derived from the training set, followed by ROC curve generation for hub genes in the validation cohort.

### GSEA analysis associated with hub gene differential expression

Samples in the integrated dataset were stratified into high-expression (high) and low-expression (low) groups based on the expression levels of hub genes. Differential expression analysis between the high and low groups was performed using the R package *limma*. GSEA was subsequently conducted on DEGs using the R package *clusterProfiler*, with significance thresholds set at an adjusted *p*-value (adj. *p*) of <0.05 and a false discovery rate (FDR) *q*-value of <0.25. The Benjamini–Hochberg (BH) method was applied for multiple testing correction.

### Construction of hub gene regulatory networks and molecular docking simulation

Transcription factors (TFs) regulating hub genes were retrieved from the ChIPBase database (http://rna.sysu.edu.cn/chipbase/). miRNAs associated with hub genes were identified through the ENCORI database (https://rnasysu.com/encori/). Direct and indirect drug targets of hub genes were predicted using the Comparative Toxicogenomics Database (CTD, https://ctdbase.org/). Three distinct regulatory networks—mRNA-TF network, mRNA-miRNA network, and mRNA-drug network—were visualized separately using Cytoscape software. The three-dimensional structures of six drug molecules—acetazolamide (CID: 1986), progesterone (CID: 5994), benzo(a)pyrene (CID: 2336), valproic acid (CID: 3121), doxorubicin (CID: 31703), and cyclosporine (CID: 5284373)—were downloaded from the PubChem database (https://pubchem.ncbi.nlm.nih.gov). X-ray crystal structures of hub genes were retrieved from the Protein Data Bank (PDB, https://www.rcsb.org/). Molecular docking between hub genes and the small-molecule compounds, along with visualization of binding modes, was performed using the AutoDock Vina program embedded in the CB-Dock2 web server (http://clab.labshare.cn/cb-dock2/).

### Consensus clustering analysis

Disease subtypes of AS samples in the training dataset based on hub gene expression profile were identified using the consensus clustering approach implemented in the R package *ConsensusClusterPlus*. Subsequently, a heatmap was generated to visualize hub gene expression patterns across distinct AS subtypes by the R package *ggplot2*. Principal component analysis (PCA) clustering patterns of AS subtypes were further characterized using the *FactoMineR* and *factoextra* packages.

### Immune infiltration analysis

AS samples in the training dataset were stratified into high-risk and low-risk groups based on the median value of the linear predictor derived from the logistic diagnostic model. The relative abundance of immune cell infiltration in each sample was quantified using the single-sample gene set enrichment analysis (ssGSEA) algorithm. Spearman correlation analysis was applied to both the immune cell infiltration matrix and the relationships between hub genes and immune cells. Immune cell infiltration levels were similarly evaluated via ssGSEA across distinct molecular subtypes [Cluster1 (C1), Cluster2 (C2)]. Differential immune infiltration between C1 and C2 groups was statistically analyzed, and correlations between hub genes and specific immune cell subsets were quantified. All results, including immune cell abundance heatmaps, correlation networks, and subtype-specific infiltration patterns, were visualized using the R package *ggplot2.*

## Results

### Identification of OCRDEGs in AS

**Figure 1:**
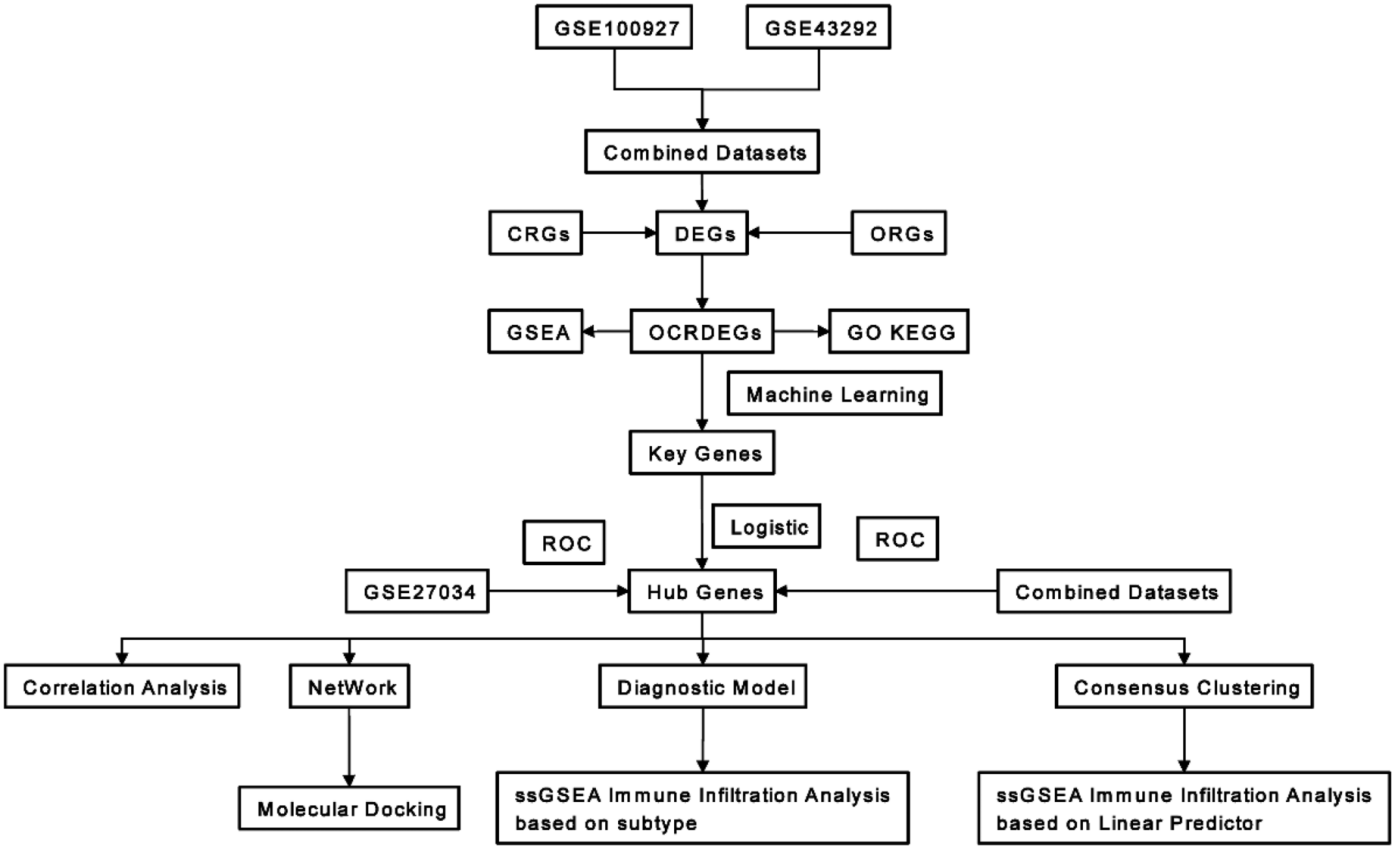
Workflow of this research.

The study workflow is summarized in Figure 1. Through systematic screening of the GeneCards database and published literature, we identified 3,192 circadian rhythm-related genes (CRGs) and 11,050 oxidative stress-related genes (ORGs) (Supplementary Tables S1, S2).

**Figure 2:**
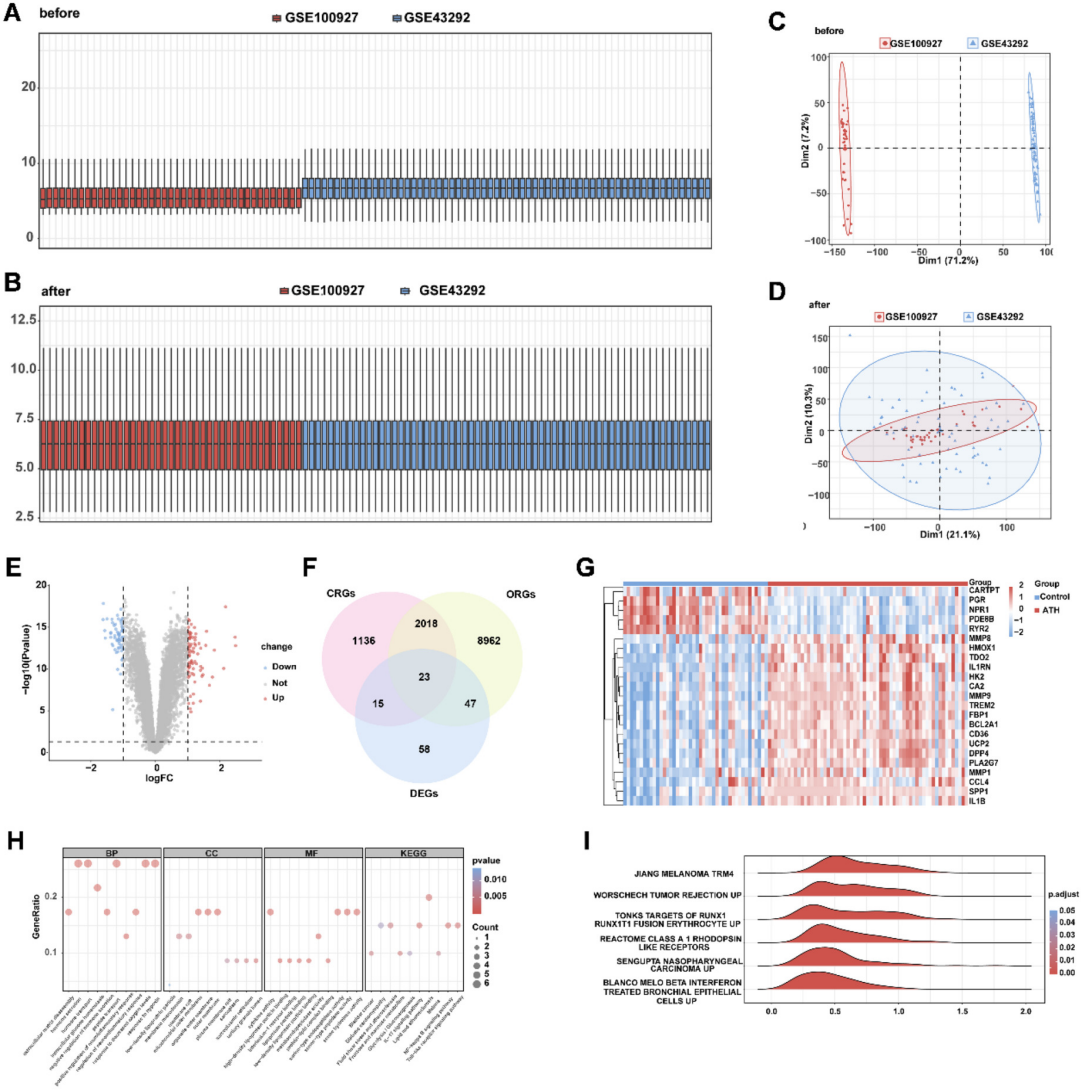
Identification of oxidative stress–circadian rhythm-related differentially expressed genes in AS and normal tissues. **(A)** Box plot of the combined dataset distribution before removing batch effects. **(B)** Box plot of the combined dataset distribution after removing batch effects. **(C)** PCA plot of the combined dataset distribution before removing batch effects. **(D)** PCA plot of the combined dataset distribution after removing batch effects. **(E)** Volcano plot of differentially expressed genes between AS and control samples in the combined dataset. **(F)** Venn diagram of differentially expressed genes and oxidative stress–circadian rhythm-related genes. **(G)** Heat map of OCRDEGs expression pattern in the combined dataset. **(H)** The GO and KEGG enrichment on OCRDEGs. **(I)** GSEA biological functional enrichment on OCRDEGs.

The batch effect removal was performed on GSE100927 and GSE43292 to obtain an integrated dataset. The expression value differences between the datasets before and after batch effect removal were analyzed (Figures 2A,B), and the low-dimensional feature distribution of the datasets before and after batch effect removal was compared using PCA (Figures 2C,D). The results indicated that the batch effects in the integrated dataset were largely eliminated after the removal process. Differential expression analysis of AS vs. control samples in the integrated dataset revealed 143 significantly differentially expressed genes (DEGs), comprising 82 upregulated and 61 downregulated genes (|log2FC| > 1.0, adjusted *p* < 0.05; Figure 2E). Intersection analysis between DEGs and OCRGs via Venn diagram identified 23 oxidative stress–circadian rhythm-related differentially expressed genes (OCRDEGs), including *FBP1*, *IL1RN*, *TREM2*, *SPP1*, *CA2*, *CARTPT*, *HMOX1*, *CD36*, *IL1B*, *HK2*, *PDE8B*, *MMP9*, *RYR2*, *PGR*, *DPP4*, *UCP2*, *BCL2A1*, *PLA2G7*, *MMP8*, *NPR1*, *TDO2*, *CCL4*, and *MMP1* (Figure 2F). Hierarchical clustering analysis demonstrated distinct expression patterns of these OCRDEGs between the AS and control groups (Figure 2G). Enrichment analysis has been used to elucidate the alteration of biological function associated with OCRDEGs. Gene Ontology (GO) enrichment analysis revealed OCRDEGs were significantly associated with biological processes (BP) (peptide transport, hypoxia response, hormone secretion, and oxygen-level regulation); cellular components (CC) (mitochondrial outer membrane and organelle outer membrane systems); and molecular functions (MF) (serine-type endopeptidase activity and cytokine signaling). KEGG pathway analysis identified “lipid and atherosclerosis” as the most significantly enriched pathway (Figure 2H). Gene set enrichment analysis (GSEA) of all DEGs demonstrated marked functional disparities between AS and control samples, particularly in targets of RUNX1 RUNX1T1 fusion erythrocyte, melanoma, β-IFN–treated bronchial epithelial cells, Class A1 rhodopsin-like receptors, and tumor rejection (Figure 2I).

### Screen of core OCRDEGs and construction of a logistic diagnostic model

**Figure 3:**
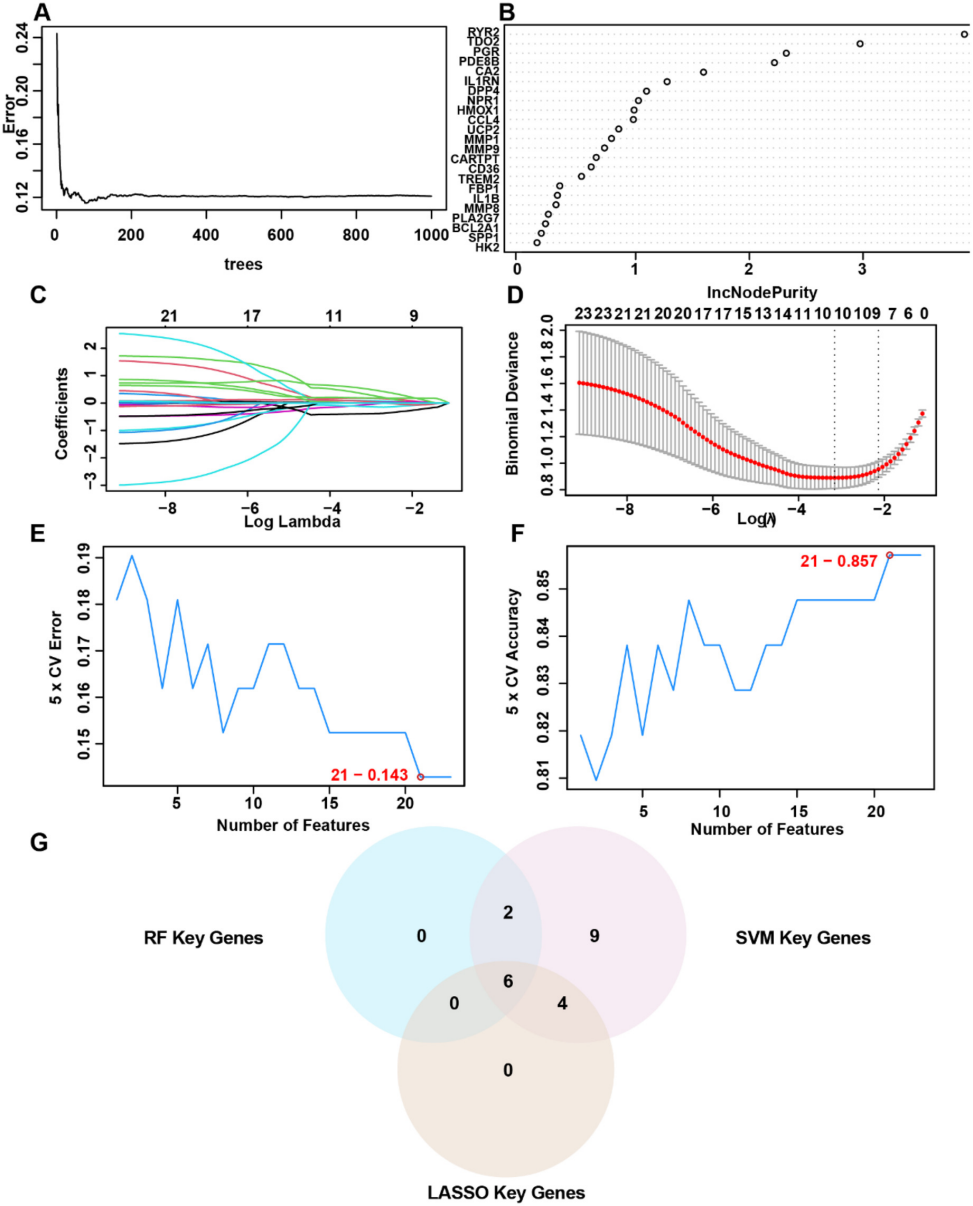
Selection of OCRDEGs-related risk genes on AS. **(A)** Decision tree error curve. **(B)** The screening results of the OCRDEGs by the random forest classifier. **(C)** Cross-validation for tuning parameter selection in the LASSO model. **(D)** LASSO regression of the 10 OCRDEGs. **(E)** The number of genes with the lowest error rate obtained by the SVM-RF algorithm. **(F)** The number of genes with the highest accurate rate obtained by the SVM-RF algorithm. **(G)** Intersection genes in the Venn diagram.

**Figure 4:**
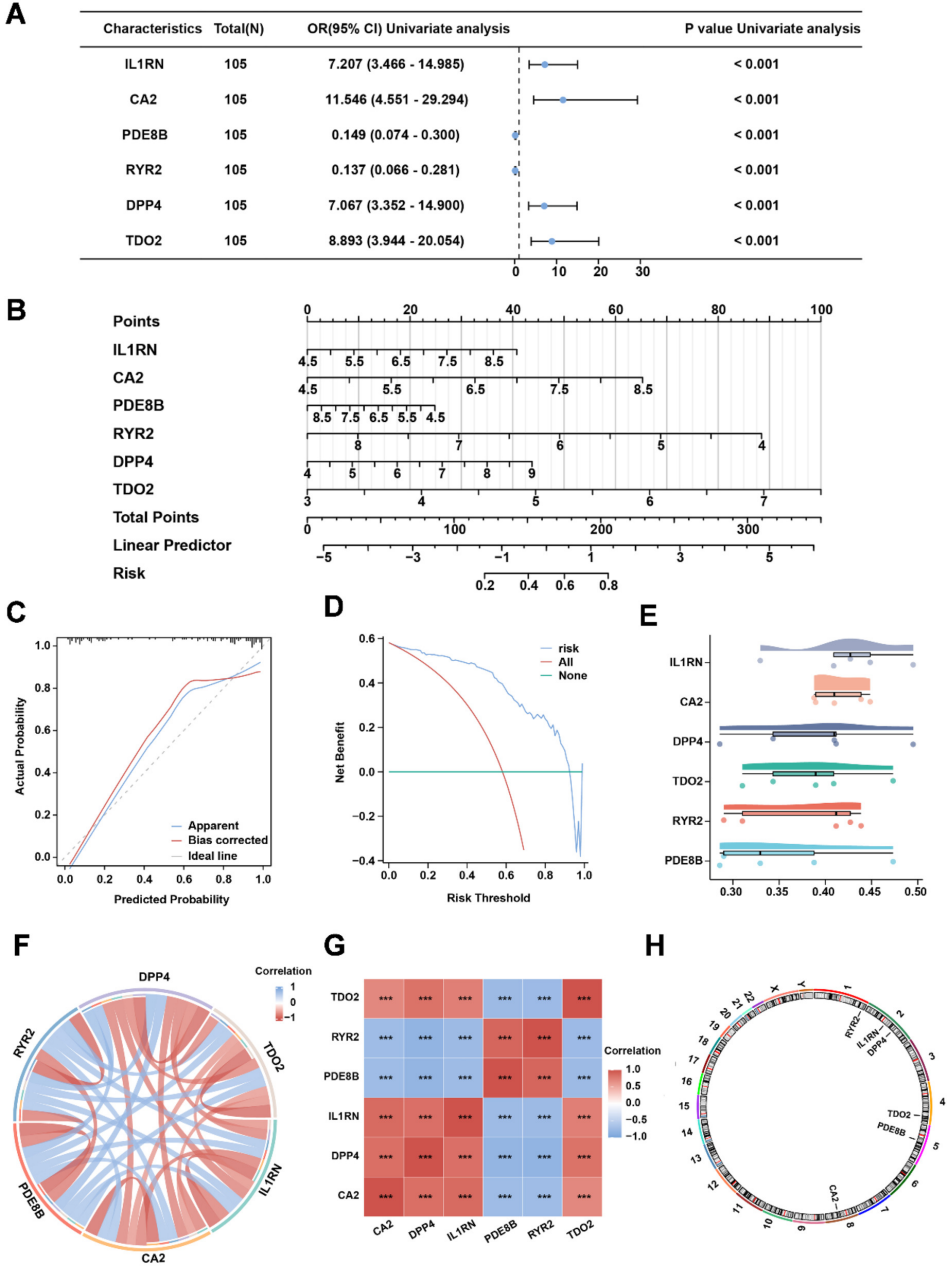
The six-risk gene model in AS. **(A)** Univariate analysis of the six risk genes. **(B)** Nomogram of risk genes in the AS diagnostic model. **(C)** Calibration curve of the AS diagnostic model. **(D)** DCA curve of the AS diagnostic model. **(E)** Friends' analysis of risk genes. **(F,G)** The correlation between each risk gene in the combined dataset. **(H)** Chromosomal location of each risk gene.

To identify AS-associated risk genes, three machine learning algorithms (random forest, LASSO regression, and SVM-RFE method) were performed on 23 OCRDEGs, and then overlapping genes were selected as characteristic risk genes. The random forest algorithm selects feature importance results based on the threshold of average node purity, visualized by plotting the decision tree error curve (Figure 3A) and the MeanDecreaseGini scatter plot (Figure 3B). Genes with IncNodePurity ≥ 1 were filtered, identifying eight of the most important genes: *IL1RN*, *CA2*, *PDE8B*, *RYR2*, *PGR*, *DPP4*, *NPR1*, and *TDO2*. LASSO regression analysis selected 10 genes (*IL1RN*, *TREM2*, *CA2*, *CARTPT*, *CD36*, *PDE8B*, *MMP9*, *RYR2*, *DPP4*, and *TDO2*) through the construction of a regression analysis model (Figure 3C) and variable trajectory plot (Figure 3D). The SVM-RFE algorithm displays the number of genes with the lowest error rate (Figure 3E) and the highest accuracy (Figure 3F) through line graphs. The results show that the SVM model achieves the highest accuracy when the number of genes is 21. These 21 OCRDEGs include *TREM2*, *CARTPT*, *PLA2G7*, *TDO2*, *DPP4*, *BCL2A1*, *CD36*, *CA2*, *HMOX1*, *PGR*, *HK2*, *IL1B*, *IL1RN*, *PDE8B*, *MMP9*, *NPR1*, *SPP1*, *RYR2*, *MMP8*, *MMP1*, and *CCL4*. Then, we screened six common genes from the above three results (Figure 3G). All six genes (*IL1RN*, *CA2*, *PDE8B*, *RYR2*, *DPP4*, and *TDO2*) demonstrated statistically significant associations (*p* < 0.05) in both univariate and multivariate logistic regression analyses, establishing them as hub genes for AS (Figure 4A). Among these genes, *PDE8B* and *RYR2* were identified as protective factors (OR < 1), while *TDO2*, *IL1RN*, *DPP4*, and *CA2* acted as risk factors (OR > 1). A nomogram diagnostic model for AS was subsequently constructed based on these hub genes (Figure 4B). The model's accuracy was validated using calibration curves, which revealed close alignment between predicted and observed probabilities (Figure 4C). Decision curve analysis (DCA) further demonstrated that the six-gene logistic model exhibited superior clinical benefit compared with “all” or “none” strategies across a defined threshold range (Figure 4D). Functional similarity scoring (friends score) highlighted *IL1RN* as the gene with the strongest correlations to other OCRDEGs, suggesting its pivotal role in AS pathogenesis (Figure 4E). Spearman correlation analysis revealed negative correlations between *RYR2*/*PDE8B* and the remaining four genes, whereas *TDO2*, *IL1RN*, *DPP4*, and *CA2* showed significant positive intercorrelations (Figures 4F,G). Then, we explored the spatial position of the abovementioned six risk genes in the genetic system. Chromosomal mapping localized *RYR2* to Chromosome 1, *IL1RN* and *DPP4* to Chromosome 2, *TDO2* to Chromosome 4, *PDE8B* to Chromosome 5, and *CA2* to Chromosome 8 (Figure 4H). The close chromosomal colocalization of these genes offers circumstantial support for their functional interconnectedness. These results collectively demonstrate the diagnostic reliability of the six-gene model for AS and underscore significant functional and positional relationships among the hub genes.

### Expression of risk genes in atherosclerosis and ROC analysis

**Figure 5:**
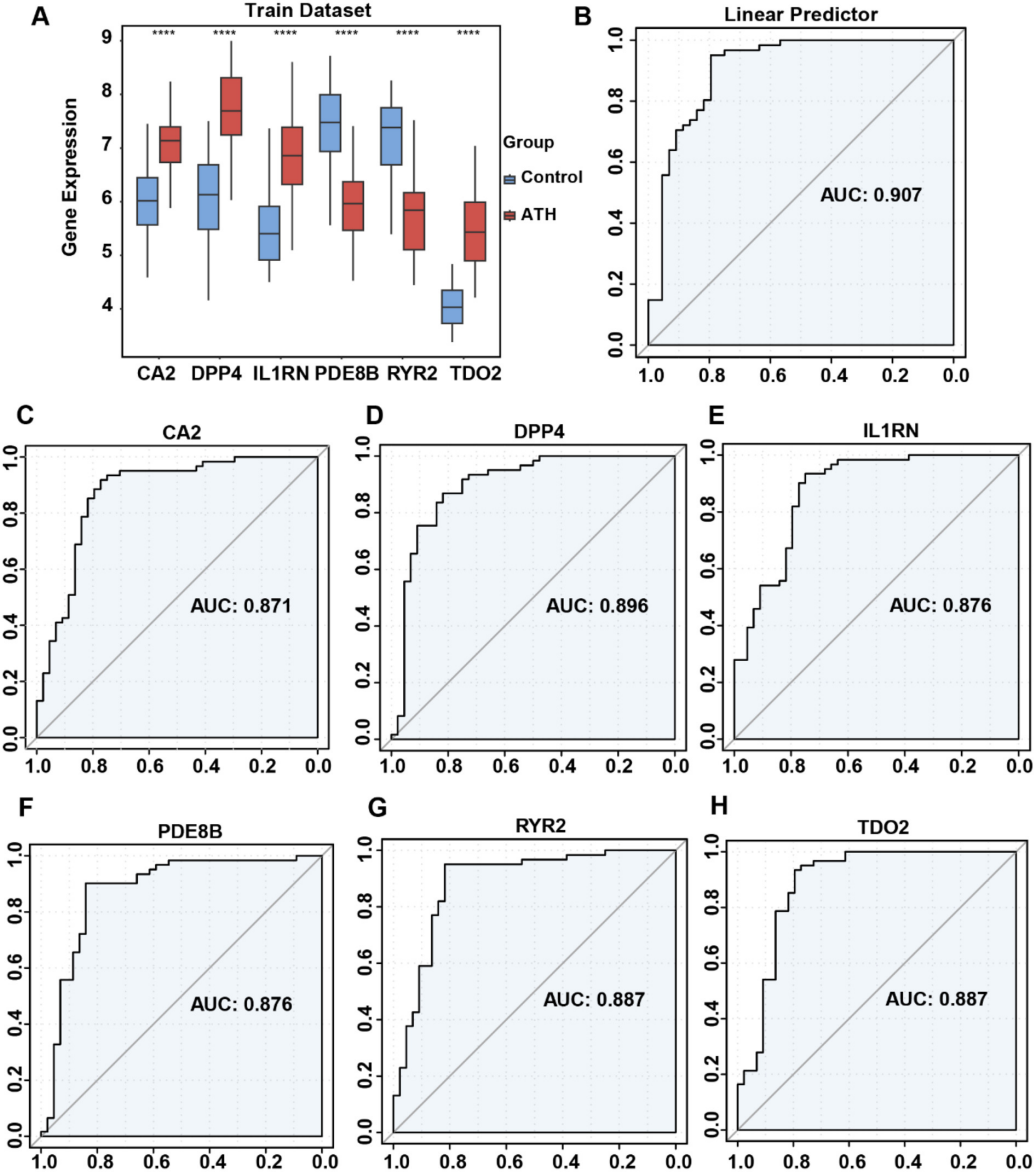
Differential expression of risk genes and ROC curve analysis of risk model in the combined dataset. **(A)** Comparison of risk gene expression between AS and normal samples. **(B)** ROC analysis of risk gene-related multivariate logistic regression model. **(C)** ROC analysis of the single *CA2* gene. **(D)** ROC analysis of the single *DPP4* gene. **(E)** ROC analysis of the single *IL1RN* gene. **(F)** ROC analysis of the single *PDE8B* gene. **(G)** ROC analysis of the single *RYR2* gene. **(H)** ROC analysis of the single *TDO2* gene.

To investigate expression differences of risk genes between AS and normal samples, we analyzed their expression profiles in both training and validation datasets. In the training dataset, *IL1RN*, *CA2*, *DPP4*, and *TDO2* were significantly upregulated in AS samples, whereas *PDE8B* and *RYR2* showed marked downregulation (Figure 5A). In the validation dataset, only *CA2* and *IL1RN* exhibited statistically significant differential expression; however, their directional trends aligned with those observed in the training dataset, potentially attributable to the smaller cohort size of the validation dataset (Supplementary Figure S1A). ROC analysis was subsequently performed to evaluate the diagnostic performance of both individual OCRDEGs and the linear predictor derived from the multivariate logistic model constructed in the training dataset. The ROC curves based on the linear predictor demonstrated a significantly higher AUC value (AUC: 0.907) compared with those of individual genes (Figure 5B). Similarly, in the validation dataset, the linear predictor achieved a larger AUC (AUC: 0.746) (Supplementary Figure S2B). These results indicate that the multivariate logistic diagnostic model outperforms single-gene predictors in accurately stratifying AS risk.

### Biological functional analysis of risk genes in AS

**Figure 6:**
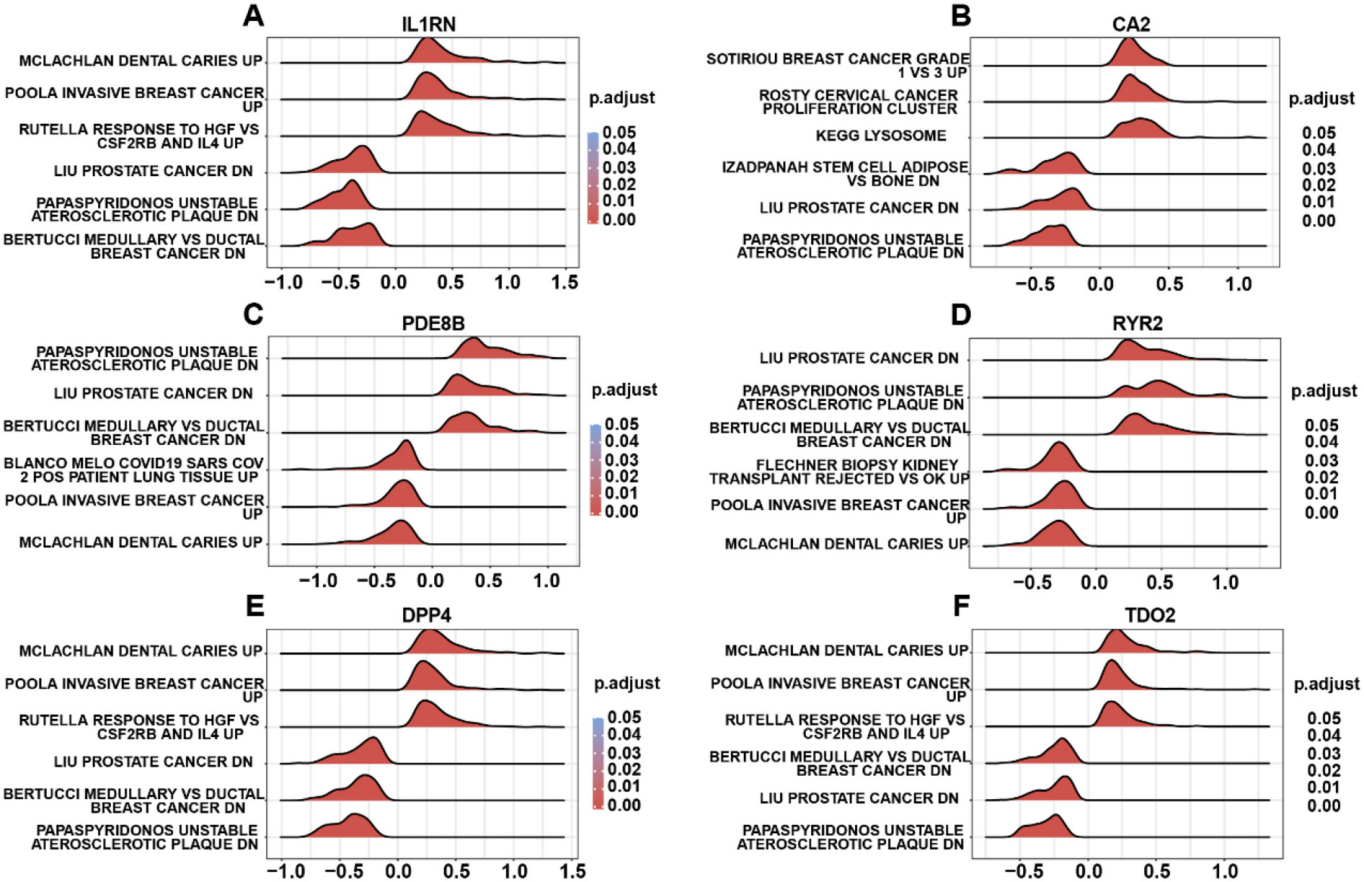
GSEA of risk gene-related DEGs. **(A)** GSEA terms of *IL1RN*-related DEGs. **(B)** GSEA terms of *CA2*-related DEGs. **(C)** GSEA terms of *PDE8B*-related DEGs. **(D)** GSEA terms of *RYR2*-related DEGs. **(E)** GSEA terms of *DPP4*-related DEGs. **(F)** GSEA terms of *TDO2*-related DEGs.

To elucidate the biological relevance of risk genes in AS, GSEA was performed on DEGs associated with risk gene expression in the integrated disease cohort. GSEA revealed significant enrichment of risk gene-related DEGs in pathways including Clachlan dental caries (Figure 6A), breast cancer grade (Figure 6B), unstable atherosclerotic plaque (Figure 6C), prostate cancer (Figure 6D), and Mclachlan dental caries (Figures 6E,F), highlighting their potential roles in AS pathogenesis. Nextly, we probed the risk gene-related regulatory map at various molecular levels. The risk gene-related regulatory networks, including miRNAs, transcription factors (TFs), and small-molecule drugs, were constructed using the ENCORI, ChIPBase, and CTD databases. The network comprised 36 TFs (Supplementary Figure S2A), 32 miRNAs (Supplementary Figure S2B), and 61 small-molecule compounds (Supplementary Figure S2C) separately, and *DPP4* emerged as the most TF-regulated gene, *CA2* as the most miRNA-targeted gene, and *IL1RN* as the most drug-responsive gene. Finally, we carried out drug target docking analysis via CB-Dock2 identified robust binding affinities between *DPP4* and progesterone, *IL1RN* and benzo(a)pyrene, and *RYR2* and doxorubicin. Moderate binding capacities were observed for *CA2*–acetazolamide and *PDE8B*–valproic acid pairs (Supplementary Figure S3). These findings delineate the biological and regulatory mechanisms of AS-associated risk genes and provide a foundational framework for targeted drug discovery.

### Immune infiltration heterogeneity between the high- and low-risk AS groups

**Figure 7:**
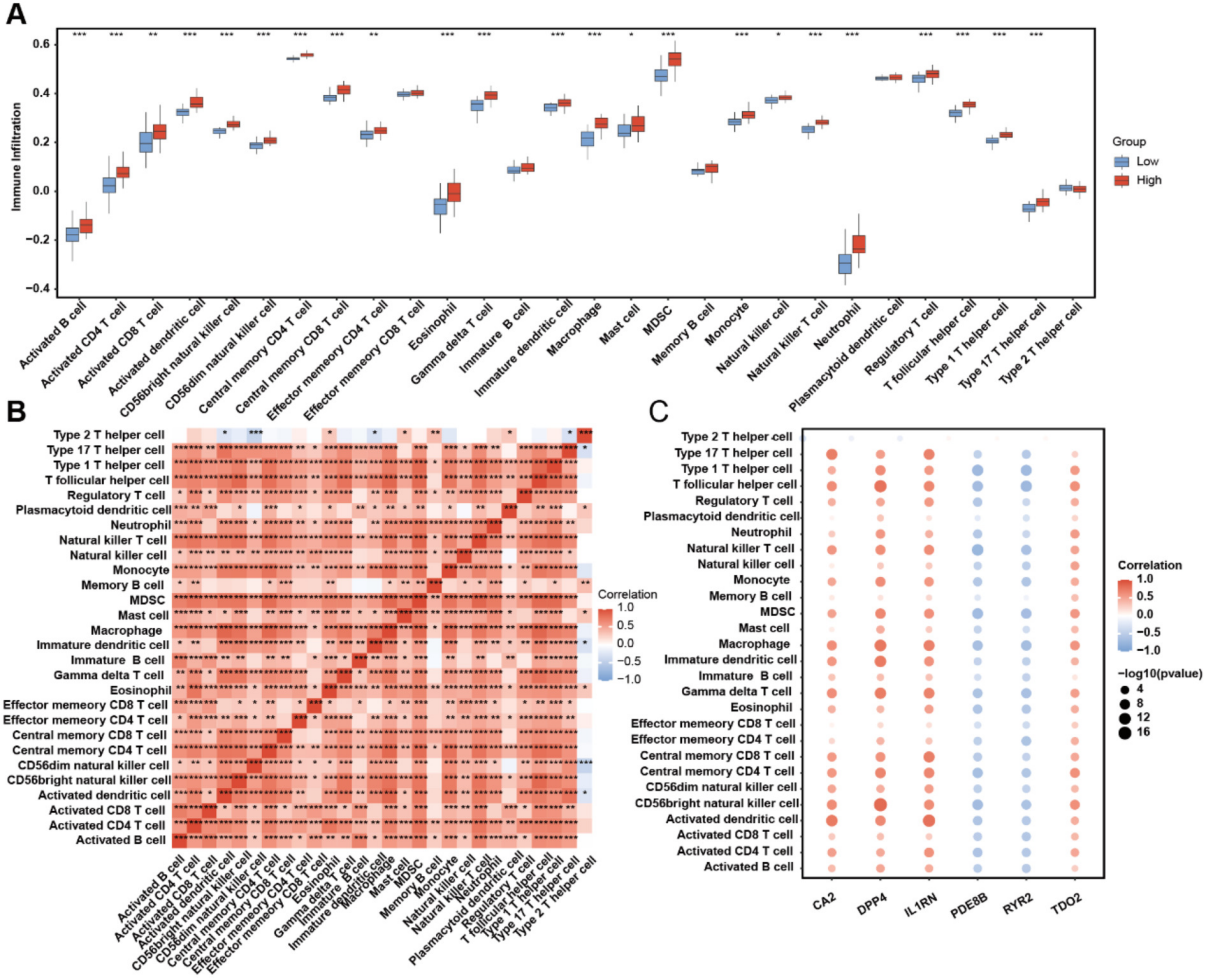
ssGSEA immune infiltration discrepancy in AS samples of different risk groups. **(A)** Immune cell infiltration abundance in the high- and low-risk AS samples. **(B)** The correlation among each immune cell type. **(C)** Correlation between risk genes and immune cell infiltration abundance.

AS samples in the training dataset were stratified into high-risk and low-risk groups based on linear predictor scores derived from the multivariate logistic model. Immune cell infiltration abundances for 28 immune cell subtypes were quantified using single-sample gene set enrichment analysis (ssGSEA) (Figure 7A). A total of 19 immune cell types—including activated B cells, activated CD4+ T cells, activated dendritic cells, CD56bright natural killer cells, CD56dim natural killer cells, central memory CD4+ T cells, central memory CD8+ T cells, eosinophils, gamma delta T cells, immature dendritic cells, macrophages, myeloid-derived suppressor cells (MDSCs), monocytes, natural killer T cells, neutrophils, regulatory T cells, T follicular helper cells, Type 1 T helper cells, and Type 17 T helper cells—exhibited significantly higher infiltration levels in the high-risk group than those in the low-risk group (*p* < 0.001). Correlation analysis of immune cell infiltration abundances revealed predominantly positive intercellular interactions across all 28 subtypes (Figure 7B). Furthermore, risk gene expression levels showed distinct immune correlation patterns: *PDE8B* and *RYR2* were inversely correlated with immune cell infiltration, whereas *CA2*, *DPP4*, *IL1RN*, and *TDO2* demonstrated robust positive correlations with most immune subtypes (Figure 7C). These findings underscore the discriminatory power of our risk stratification model in identifying immune infiltration patterns in AS. The elevated immune infiltration observed in high-risk patients suggests that dysregulated immunomodulatory mechanisms are associated with AS development in high-risk patients.

### Consensus clustering analysis based on risk genes

**Figure 8:**
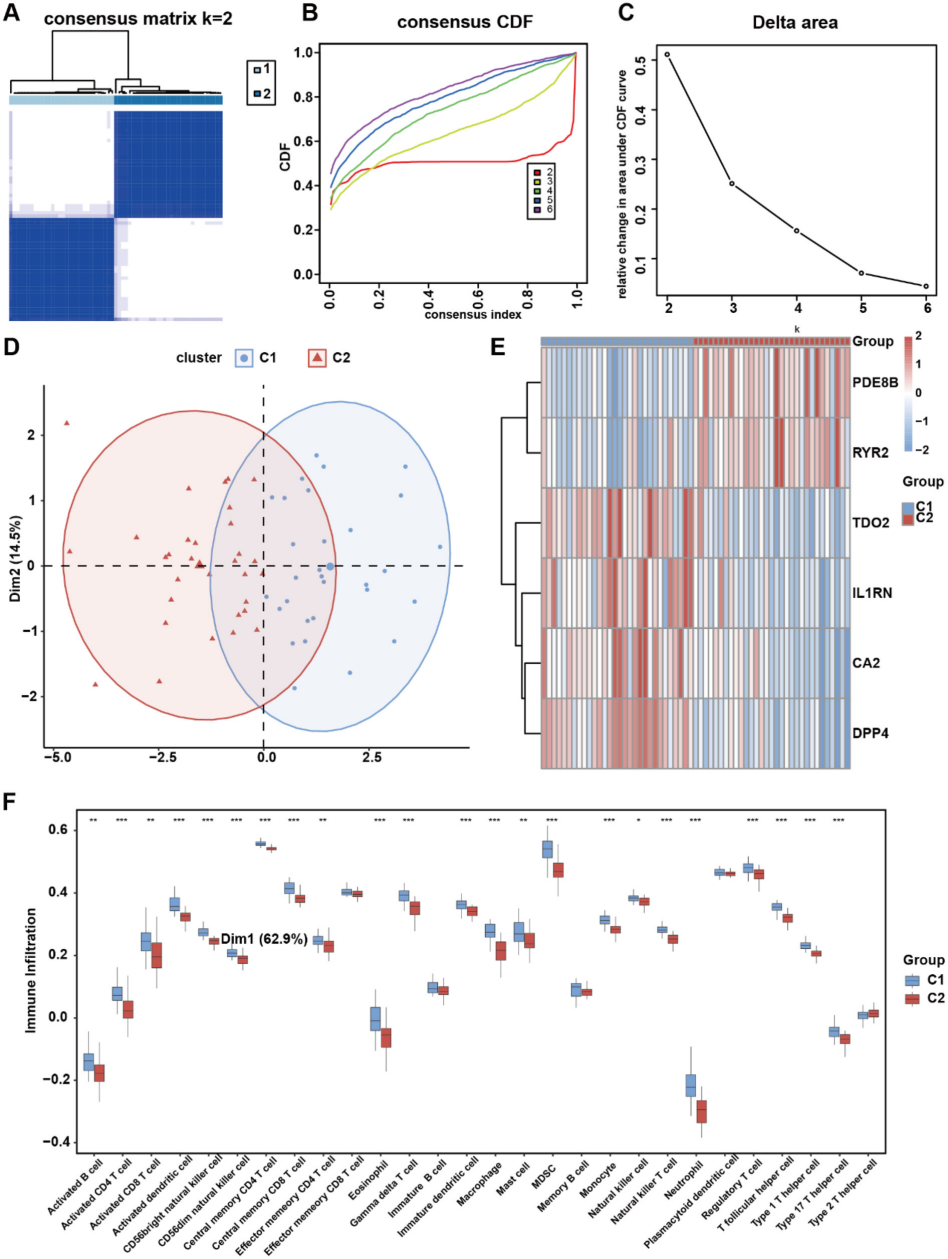
OCRDEG risk gene molecular subtype clustering analysis. **(A)** Consensus clustering of AS samples according to the risk gene expression background. **(B)** Consensus clustering cumulative distribution function (CDF) for *k* = 2–6. **(C)** The delta area score determines the stability of clustering. **(D)** PCA clustering plot of two AS subtypes. **(E)** Risk gene expression pattern in different AS subtypes. **(F)** The difference in immune cell infiltration in the two AS subtypes.

Unsupervised clustering can categorize patients into subgroups with different molecular characteristics, and patients in different subgroups may have varying prognostic features (36). Therefore, clustering analysis is widely used in the research of various diseases. To investigate potential expression patterns of the six risk genes in AS, consensus clustering analysis was performed on the integrated AS cohort to identify disease subtypes associated with risk gene expression profiles. We carried out unsupervised consensus clustering analysis based on *IL1RN*, *CA2*, *PDE8B*, *RYR2*, *DPP4*, and *TDO2* expression levels to categorize all samples into two groups. The results indicated that *k* = 2 might be an optimal selection for clarifying patients into two groups, including molecular Cluster 1 (*n* = 29) and Cluster 2 (*n* = 31) (Figures 8A–C). Principal component analysis (PCA) confirmed significant molecular heterogeneity between these subtypes (Figure 8D). Differential expression analysis revealed subtype-specific risk gene expression patterns: *PDE8B* and *RYR2* were significantly upregulated in Cluster 2, while *TDO2*, *IL1RN*, *CA2*, and *DPP4* showed higher expression in Cluster 1 (Figure 8E). Subsequent immune infiltration analysis demonstrated markedly lower infiltration levels of 19 immune cell types—including activated B cells, activated CD4+ T cells, activated dendritic cells, CD56bright natural killer cells, CD56dim natural killer cells, central memory CD4+ T cells, central memory CD8+ T cells, eosinophils, gamma delta T cells, immature dendritic cells, macrophages, myeloid-derived suppressor cells (MDSCs), monocytes, natural killer T cells, neutrophils, regulatory T cells, T follicular helper cells, Type 1 T helper cells, and Type 17 T helper cells—in Cluster 2 than those in Cluster 1 (*p* < 0.001, Figure 8F). These results collectively demonstrate that risk genes exhibit coordinated expression patterns capable of stratifying AS patients into molecularly distinct subtypes, which are further characterized by divergent immune microenvironment landscapes.

## Discussion

As a worldwide burden for human health, AS leads to multiple clinical symptoms, including hypertension, coronary heart disease, cerebrovascular disease, peripheral vascular disease, heart failure, rheumatic heart disease, congenital heart disease, and cardiomyopathies (37, 38). Oxidative stress, the imbalance between the reactive oxygen species and the intracellular antioxidative protection system, often originates from the redundancy of ROS or the invalidation of the reducing system (39). Its occurrence is closely related to a lot of disorders such as tumors, coronary heart disease, and neurological disorders (40–43). ROS takes multiple roles across atherosclerosis from start to finish and is regarded as a significant characteristic of AS (44). These previous conclusion illustrates there is prospective potential to obtain excellent clinical benefits from AS treatment through targeting oxidative stress in different AS stages. The strong correlation between AS and oxidative stress also hints us a few oxidative stress-related genes that could act as diagnostic markers and the basis for identifying the molecular background in AS. In recent years, researchers have gradually recognized the critical role of circadian rhythms in the development of AS. Disruption of circadian rhythms promotes the progression of atherosclerosis. For example, incorrect lighting conditions can lead to a disturbance of the biological clock, which in turn affects the function of endothelial cells and increases inflammation responses and oxidative stress levels, leading to the exacerbation of AS (45). Circadian rhythms also regulate the interaction between immune cells and blood vessels, influencing the occurrence and development of inflammation (22). This evidence shows the significant correlation between circadian rhythms and oxidative stress in atherosclerosis. Therefore, we resorted to bioinformatic methods to identify the oxidative stress–circadian rhythm-related diagnostic model of AS to reveal their interaction mechanism.

Among the 23 differentially expressed OCRDEGs identified in this work, *IL1RN*, *CA2*, *PDE8B*, *RYR2*, *DPP4*, and *TDO2* were closely associated with AS and could act as AS-related risk genes. *IL1RN* is involved in encoding the interleukin-1 receptor antagonist, playing an indispensable role in regulating inflammatory responses, and its polymorphisms are closely related to susceptibility to various diseases (46). *CA2* encodes a member of the carbonic anhydrase family, which is involved in regulating intracellular and extracellular pH levels as well as carbon dioxide elimination (47). *PDE8B* belongs to the phosphodiesterase family and is primarily responsible for the degradation of cyclic adenosine monophosphate (cAMP) and cyclic guanosine monophosphate (cGMP). Dysregulation of *PDE8B* is associated with the onset of various diseases, including cardiovascular diseases, neurological disorders, and metabolic diseases (48–50). *RYR2* encodes a key calcium ion channel that plays a role in the contraction of cardiac muscle cells (51). *DPP-4* is an important endogenous enzyme that is widely present in various tissues, regulating insulin secretion by degrading insulinotropic hormones such as GLP-1 and GIP (52). *TDO2* encodes tryptophan-2,3-dioxygenase (TDO), which plays a key role in tryptophan metabolism. Recent studies have gradually revealed a close relationship between *TDO2* and various neuropsychiatric disorders, particularly in the pathogenesis of depression, anxiety, and other mood disorders (53). At the same time, the overexpression of *TDO2* is closely related to the occurrence and development of various cancers, especially showing significant prognostic value in malignant tumors such as hepatocellular carcinoma and lung adenocarcinoma (54).

Through our analyses, the abovementioned six genes could be considered as diagnostic markers of AS, and we also validated the diagnostic efficacy by other methods. ROC curves exhibited the AUC of *IL1RN*, *CA2*, *PDE8B*, *RYR2*, *DPP4*, and *TDO2*, and the multivariate diagnostic model combined these genes. The AUC of six risk genes is much higher than a single-gene diagnostic model that validates the reliability of our six-risk gene model. Despite the model's AUC value in the training set and validation set both being >0.700, the model's performance in the training set is higher than that in the validation set (0.907 vs. 0.746). As a pivotal limitation, the insufficient sample size of patients may lead to a decline in model performance. In addition, this phenomenon may be attributed to batch effects caused by the different ways sequencing platforms handle samples, as well as overfitting of the model in the training dataset. The issues of overfitting include decreased validation performance, reduced biological interpretability, and poor cross-platform generalization ability. When the model is too complex or data preprocessing is inadequate, using too many features or failing to apply appropriate regularization can cause the model to capture noise in the data rather than the true signal (55). In the future, we will build a database that includes more clinical samples. Based on a broader range of clinical samples, we will enhance the model's effectiveness through regularization, cross-validation, simplification of model structure, and biological methods.

Enrichment analyses of each risk gene-related DEG revealed the difference in biological process. Different regulatory networks showed us a relatively comprehensive molecular profile regarding risk genes, including transfactor level or transcript level. In addition, the drug–gene interaction network came up with the prospective possibility of novel targets of AS. Most significant is that we elucidated the apparent preference in immune cell infiltration between the high- and low-risk groups. Patients in the high-risk group express abundant immune cell infiltration levels that are consistent with the explicit conclusion that immune progression plays a central role in the pathogenesis of AS. The role of immune cells in atherosclerosis is multifaceted. Immune cells, including monocytes, macrophages, T cells, and B cells, can regulate local inflammatory responses by releasing cytokines and chemokines. These cytokines not only promote the inflammatory response in the arterial intima but also affect the proliferation and migration of smooth muscle cells, thereby accelerating the progression of atherosclerosis (56). Different types of immune cells play distinct roles at various stages of atherosclerosis. For example, macrophages are involved not only in the phagocytosis of lipids but also play a key regulatory role in the inflammatory microenvironment. The activation state of macrophages (such as M1 and M2 types) directly influences the progression and stability of atherosclerosis (57). At the same time, T cells, especially CD4+ and CD8+ T cells, also play important roles in atherosclerosis. Research shows that specific T-cell subsets, such as Th1 cells and regulatory T cells (Treg), have dual roles in inflammation and immune regulation, potentially affecting the progression and stability of atherosclerosis (58). Besides, there is sufficient evidence indicating the role of risk genes in AS's immune imbalance. *IL1RN* competitively inhibits the binding of IL-1α/β to receptors, blocking the activation of the NF-κB inflammatory pathway and reducing M1 polarization of macrophages and differentiation of TH17 cells (59). *RYR2* regulates T-cell calcium signaling, and gain-of-function mutations lead to increased IL-2 secretion in CD4+ T cells, which is a novel immune cell infiltration-related biomarker in atherosclerosis diagnosis (60). *DPP4* improved atherosclerosis formation in mice by inhibiting oxidative stress and can also alleviate atherosclerosis by promoting M2 macrophage polarization (61). *TDO2* consumes tryptophan through the kynurenine pathway, promoting *TH17* differentiation and inhibiting Treg function (62). Two subtypes of AS clustered by interior expression pattern between risk genes exhibit a significant distinction in immune activity status. However, the systematically direct regulatory mechanism of oxidative stress–circadian rhythm-related risk genes on the immune background of AS requires further exploration to demonstrate the intricate cross talk between the occurrence of oxidative stress and atherosclerosis development.

Although imaging-based methods are the gold standard for the diagnosis of AS, our work remains practically significant for the risk stratification of patients with atherosclerosis. The risk gene model constructed in this study can assess patients' risk based on the gene expression profiles of the affected tissues, thereby formulating individualized monitoring or treatment plans based on risk stratification, while also analyzing the differences in responses to specific treatment regimens among different risk groups. With the development of vascular surgery and the popularization of sequencing technology, the cost of risk gene stratification for plaques in atherosclerotic patients will undoubtedly be greatly reduced. Combined with traditional imaging, atherosclerotic patients will gain greater clinical benefits in future diagnostic and therapeutic practices. In conclusion, we identified six oxidative stress–circadian rhythm-related risk genes which have clinical implications, conducted a relevant AS risk model, examined the potential clinical outcome, and revealed the molecular regulation background of risk genes. These results reveal the integral viewpoint of the integrative roles of oxidative stress and circadian rhythm in the clinical hazard of AS and offer a perspective on the treatment of AS targeting redox imbalance and anomalous circadian rhythm oscillation.

## Data Availability

Publicly available datasets were analyzed in this study. Data can be found here: https://www.ncbi.nlm.nih.gov/geo/; GSE100927, GSE43292, GSE27034.
