# Supplementary material for: An oxidative stress-related molecular signature in atherosclerosis: identification of risk genes, construction of a diagnostic model, and characterization of immunocyte landscape
Source: Front Cardiovasc Med. 2025 Aug 14;12:1600321. doi: 10.3389/fcvm.2025.1600321 (PMC12392417; doi:10.3389/fcvm.2025.1600321)
Supplement: Supplementary file 1 [file Datasheet1.docx]

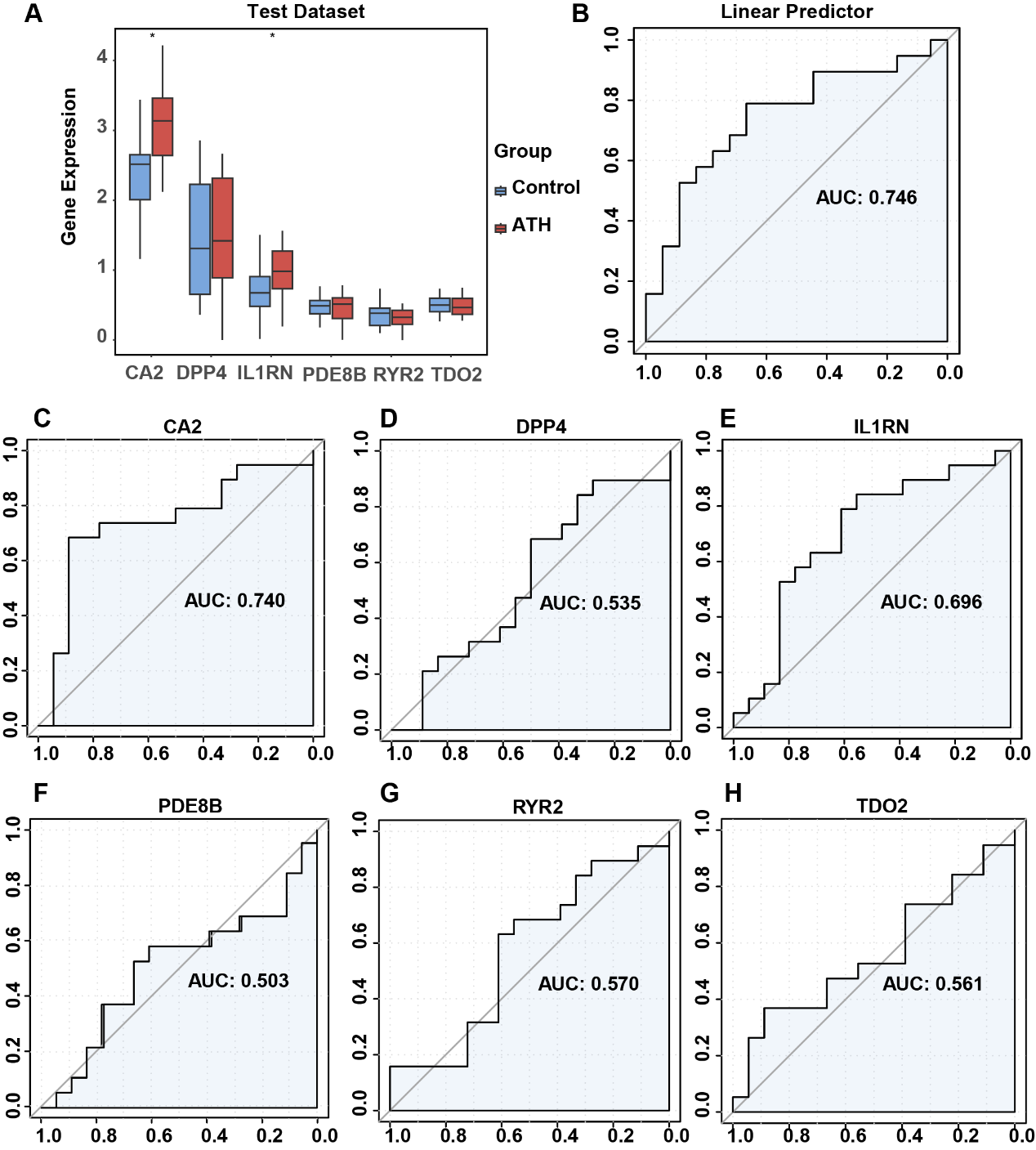


Supplementary Fig. 1 Differential Expression of risk genes and ROC Curve Analysis of risk model in validation dataset. (A) Comparison of risk genes expression between ATH and normal samples. (B) ROC analysis of risk genes -related multivariate logistic regression model. (C) ROC analysis of single *CA2* gene. (D) ROC analysis of single *DPP4* gene. (E) ROC analysis of single *IL1RN* gene. (F) ROC analysis of single *PDE8B* gene. (G) ROC analysis of single *RYR2* gene. (H) ROC analysis of single *TDO2* gene.


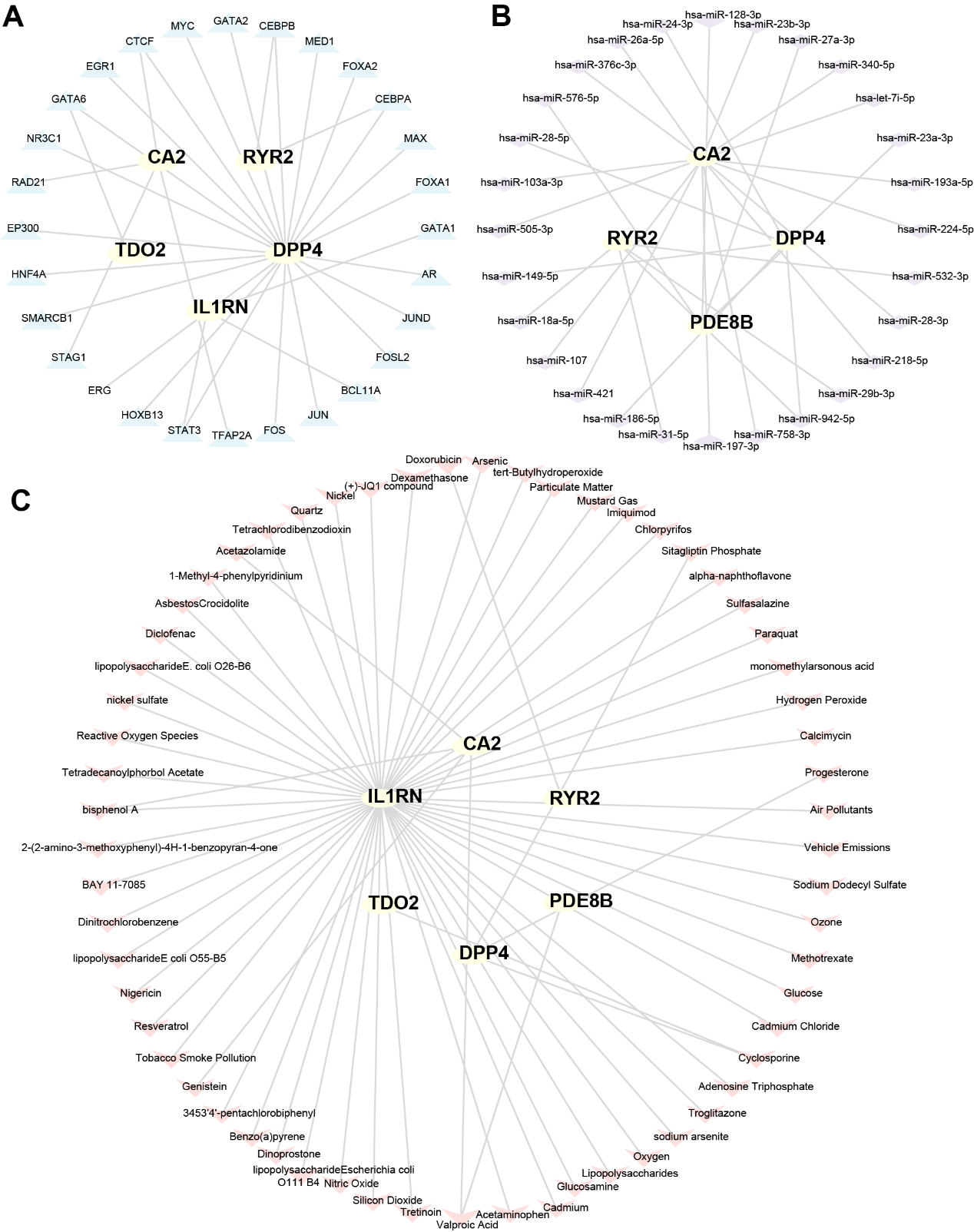


Supplementary Fig. 2 Risk genes-related regulatory network. (A) Potential risk genes-transfactors regulatory network. (B) Potential risk genes-miRNA regulatory network. (C) Potential risk genes-drug interaction network.


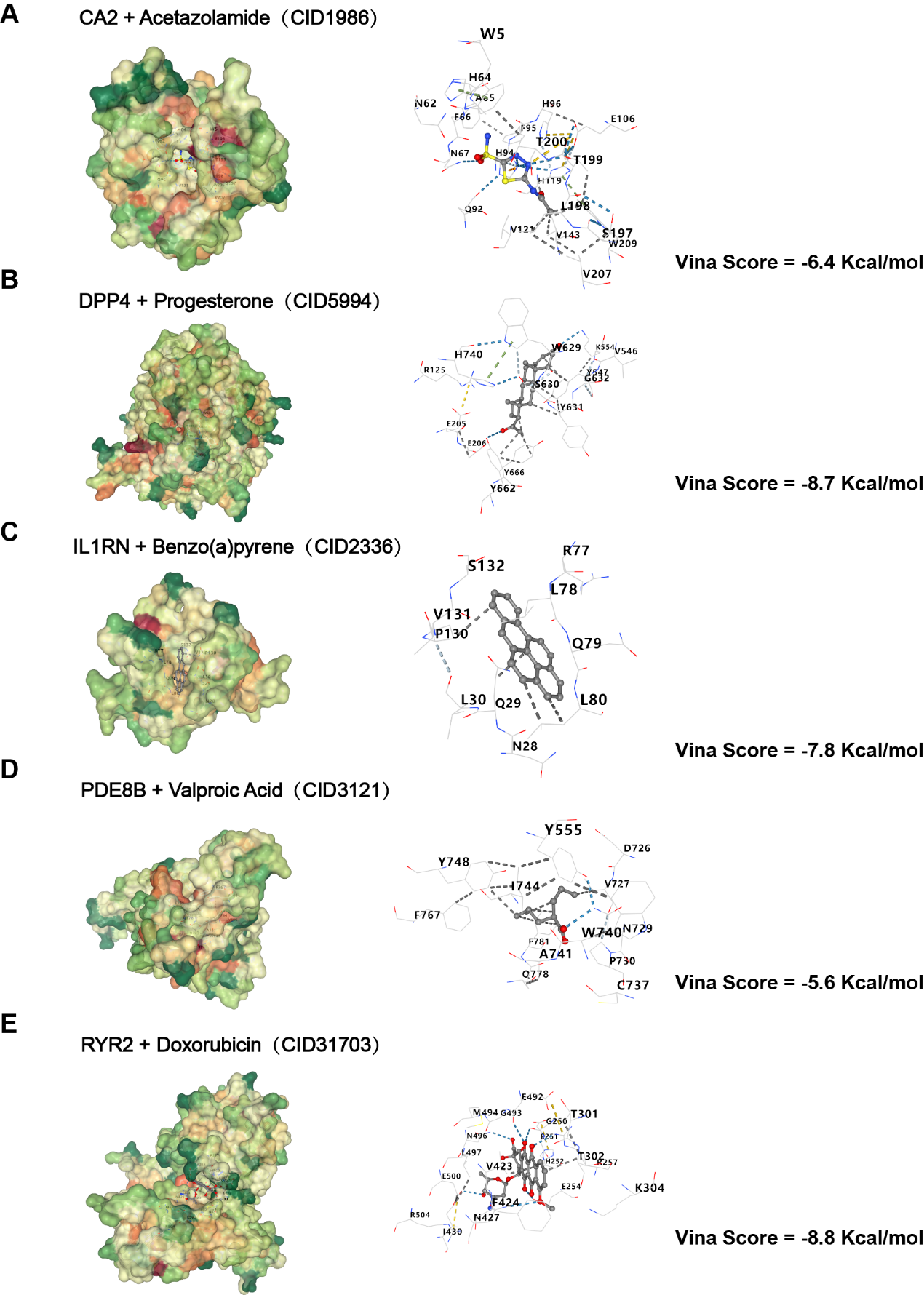


Supplementary Fig. 3 Molecular docking simulation of risk genes and small compound drugs. (A) The interaction effect simulation between CA2 and Acetazolamide. (B) The interaction effect simulation between DPP4 and Progesterone. (C) The interaction effect simulation between IL1RN and Benzo(a)pyrene. (D) The interaction effect simulation between PDE8B and Valproic Acid. (E) The interaction effect simulation between RYR2 and Doxorubicin.
